# Intention to Use Behavioral Health Data From a Health Information Exchange: Mixed Methods Study

**DOI:** 10.2196/26746

**Published:** 2021-05-27

**Authors:** Randyl A Cochran, Sue S Feldman, Nataliya V Ivankova, Allyson G Hall, William Opoku-Agyeman

**Affiliations:** 1 Department of Health Sciences College of Health Professions Towson University Towson, MD United States; 2 Department of Health Services Administration School of Health Professions University of Alabama at Birmingham Birmingham, AL United States; 3 School of Health and Applied Human Sciences College of Health and Human Services University of North Carolina Wilmington Wilmington, NC United States

**Keywords:** behavioral health, integrated care, health information exchange, behavioral intention, patient care, mixed methods research

## Abstract

**Background:**

Patients with co-occurring behavioral health and chronic medical conditions frequently overuse inpatient hospital services. This pattern of overuse contributes to inefficient health care spending. These patients require coordinated care to achieve optimal health outcomes. However, the poor exchange of health-related information between various clinicians renders the delivery of coordinated care challenging. Health information exchanges (HIEs) facilitate health-related information sharing and have been shown to be effective in chronic disease management; however, their effectiveness in the delivery of integrated care is less clear. It is prudent to consider new approaches to sharing both general medical and behavioral health information.

**Objective:**

This study aims to identify and describe factors influencing the intention to use behavioral health information that is shared through HIEs.

**Methods:**

We used a mixed methods design consisting of two sequential phases. A validated survey instrument was emailed to clinical and nonclinical staff in Alabama and Oklahoma. The survey captured information about the impact of predictors on the intention to use behavioral health data in clinical decision making. Follow-up interviews were conducted with a subsample of participants to elaborate on the survey results. Partial least squares structural equation modeling was used to analyze survey data. Thematic analysis was used to identify themes from the interviews.

**Results:**

A total of 62 participants completed the survey. In total, 63% (n=39) of the participants were clinicians. Performance expectancy (*β*=.382; *P*=.01) and trust (*β*=.539; *P*<.001) predicted intention to use behavioral health information shared via HIEs. The interviewees (n=5) expressed that behavioral health information could be useful in clinical decision making. However, privacy and confidentiality concerns discourage sharing this information, which is generally missing from patient records altogether. The interviewees also stated that training for HIE use was not mandatory; the training that was provided did not focus specifically on the exchange of behavioral health information.

**Conclusions:**

Despite barriers, individuals are willing to use behavioral health information from HIEs if they believe that it will enhance job performance and if the information being transmitted is trustworthy. The findings contribute to our understanding of the role HIEs can play in delivering integrated care, particularly to vulnerable patients.

## Introduction

### Background

Behavioral health conditions adversely affect an individual’s well-being, quality of life, and life expectancy. Behavioral health conditions are defined as mental illnesses (eg, depression, anxiety, schizophrenia, and bipolar disorder) and substance use disorders that impair the functioning of an individual [1]. Patients with these conditions require the delivery of team-based care to achieve optimal health outcomes [2,3]. Nearly half of the adult population will meet the criteria for a mental illness during their lifetime [4]. Recent projections indicate that in the United States, approximately 19% of the adult population may have a mental illness at any point in time [5-7]. Behavioral health conditions are expected to surpass chronic medical conditions to become the leading cause of disability worldwide [8], with depression alone projected to become the second most prevalent cause of disability [7].

The relationship between physical health and mental health is complex [9-11]. Patients diagnosed with a behavioral health condition frequently have chronic medical comorbidities [4,5,12-15]. The association of mental illness with chronic disease contributes to the inefficient use of health care services (eg, increased inpatient hospital utilization) [5,16-18]. Other factors such as infrequent use of primary care and preventive services, poor chronic disease management, and the use of antipsychotic medications [19-23] also contribute to the overutilization of hospital services. Patients with behavioral health conditions, coupled with chronic medical comorbidities, contribute substantially to health care spending. A population-based cohort study in Alberta, Canada, revealed that patients with a chronic medical illness and a co-occurring mental health condition had a higher mean of 3-year adjusted health care costs (Can $38,250 [US $31,700]) than chronically ill patients without a mental health condition (Can $22,250 [US $18,440]) [24]. A similar pattern is expected in the United States. In fact, the costs are likely to be higher [25]. The costs alone indicate that it is both financially and clinically prudent to consider new ways to manage behavioral health conditions in chronically ill patients [25]. A novel approach to control costs is a coordinated care delivery system.

Behavioral health patients require coordinated care but often fail to receive it [17,26]. This level of care requires better communication and information sharing between general medical and behavioral health care providers. Therefore, it is necessary to develop new approaches that facilitate the effective treatment of both medical and behavioral health conditions [27]. Health information exchanges (HIEs), a component of health information technology (IT), can facilitate the integration of different health care services provided to behavioral health patients with chronic conditions.

HIEs are organizations that enable the digital exchange of health-related data [28], and they are one type of health IT thought to facilitate such integration. The exchange of health information through HIEs has many benefits, including safer, more efficient care that effectively manages both the behavioral and physical health needs of individual patients [13,29-31]. HIEs lead to efficient information sharing between providers in emergencies that require prompt, accurate diagnosis and treatment [29]. The effectiveness of HIEs has been studied in care coordination in general, especially in chronic disease management, but there are few studies that examine HIEs in the context of behavioral health [6,32-35]. The dearth of studies in behavioral health and HIE is a result of rising challenges with behavioral health data. Specifically, there is a disjoint between the clinical language, codes, and data reporting between behavioral health and general medicine among providers [6]. In addition, federal regulations complicate behavioral health information sharing. One of these regulations, that is, Section 42 Part 2, was adopted in the Code of Federal Regulations (42 CFR Part 2) in 1972 to ensure privacy protection for individuals receiving treatment for substance use disorders. It prohibited the disclosure of records related to substance use treatment without express authorization from the patient (with certain exceptions in emergency situations) [36]. Revisions of 42 CFR Part 2 in 2017 and 2018 have aimed to modernize the regulation and to facilitate the integration of behavioral health in general medical settings. Despite these efforts, challenges remain [6,36]. Taken together, these factors create barriers to exchanging behavioral health information and delivering integrated care for this patient population.

Therefore, the aim of this study is to identify and describe various factors that may influence health care providers’ intention to use and actual use of behavioral health information obtained from an HIE. This is accomplished by using a mixed methods approach guided by the theoretical backdrop of the unified theory of acceptance and use of technology (UTAUT) and diffusion of innovation (DOI). This study addresses the following research questions:

1. What factors are associated with the intention to use behavioral health information obtained from HIEs?
2. From the perspective of health care providers, what are the facilitators of and barriers to behavioral health information use from HIEs?

### Literature Review and Conceptual Framework

Integrated behavioral health care has demonstrated effectiveness in treating patients with behavioral health disorders and co-occurring medical conditions [37,38]. Such models of care delivery can improve the quality of care by delivering treatments that align with the clinical needs of patients [38]. A systematic review revealed that among 4 randomized controlled trials, 2 of the studies showed minor improvements in physical health, whereas the other 2 studies showed no significant changes in physical health [39]. Furthermore, improvements in seeking preventive care were also observed. However, none of these studies examined the effect of these interventions on functional or clinical outcomes [39]. Improvements in both access and quality of care have been attributed to communication and information sharing between clinicians, particularly when treating patients with complex medical and behavioral needs [40]. This finding suggests that establishing effective communication channels is crucial for the success of integrated care delivery models. However, there are barriers to communication between medical and behavioral health care providers.

A 2011 study examined the perspectives of behavioral health care providers regarding the benefits and barriers of HIE utilization [41]. According to the study, behavioral health care providers stated that the use of HIEs would (1) improve the quality of care and communication between different types of providers, (2) increase cost and time burdens, (3) raise concerns related to access to and vulnerability of patient and client information, and (4) impact workflow and control. Similarly, a 2019 qualitative study explored the perspectives of patients with mental health conditions to gain an understanding of privacy in the context of exchanging personal health information [42]. All 14 participants acknowledged the importance of privacy in health care, particularly concerning information related to sensitive diagnoses (eg, mental health conditions). However, the degree of concern varied and was most frequently related to negative experiences and perceptions related to privacy. The interviewees expressed that, overall, they trusted that health care professionals and organizations would take appropriate measures to protect the privacy of information related to mental health diagnoses. The participants were largely uninformed about existing policies and regulations designed to protect the privacy of mental health information, and those who had negative experiences related to privacy expressed doubt about the effectiveness of these regulations. However, the participants acknowledged that the electronic exchange of health information was a practical approach to ensuring high-quality patient care. It is important to note that this study was conducted in Canada, where a single-payer system exists.

The 2 theories that are deemed appropriate to explore our overall research questions are the UTAUT and the DOI theory.

UTAUT integrates constructs from earlier IT adoption theories to examine their influence on individuals’ intentions to use technology and the subsequent use of the technology [43]. UTAUT explains nearly 70% of the variance related to the intention to use technology [44]. The original UTAUT model attributes behavioral intention and subsequent technology use to 4 main constructs: (1) *performance expectancy* (perceptions of the ability of the technology to assist in meeting job-related goals), (2) *effort expectancy* (ease of use of technology), (3) *social influence* (perception that important figures support the use of technology), and (4) *facilitating conditions* (infrastructure that supports the use of technology) [43,45]. Extensions of the UTAUT model have considered the influence of additional factors on behavioral intention to use technology, such as trust and perceived risk [46].

**Figure 1:**
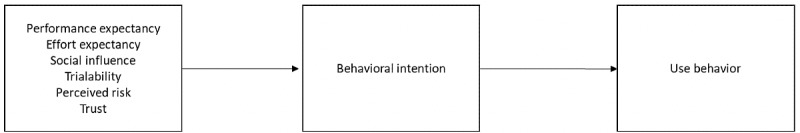
Conceptual model.

DOI examines 5 factors that determine the rate of adoption of a new technology (innovation) within an organization: (1) *relative advantage* (the innovation is perceived as a better solution than its predecessor), (2) *compatibility* (the innovation fits with the organization’s culture and norms), (3) *complexity* (the innovation is easy to understand and use), (4) *trialability* (individuals can experiment with innovation before adoption), and (5) *observability* (the results of using the innovation are visible to others) [47,48]. The trialability construct was extracted from this theory because it was not captured in the UTAUT framework. The exchange of behavioral health data via an HIE is a relatively novel concept, and it is possible that pilot testing this type of data exchange could influence behavioral intention. The conceptual model for this study, which is derived from these 2 theories, is shown in Figure 1. The research questions were identified using this model.

## Methods

### Overview

A sequential explanatory mixed methods design was used in this study. This mixed methods design enables researchers to provide more thorough explanations of the trends that emerge from the quantitative phase of the study by further exploring participants’ perspectives [49,50]. The quantitative and qualitative study phases are described in the following sections.

### Quantitative Phase

#### Sampling

Survey participants were selected through convenience sampling. Alabama and Oklahoma were selected for participation because both these states have established HIEs and reported making some efforts to incorporate behavioral health data into patient records. General medical care providers (physicians, nurses, and physician assistants) and nonclinical staff (directors, network administrators, and patient experience representatives) were identified as potential participants.

#### Data Collection

A validated survey instrument was emailed to clinicians and staff in Alabama and Oklahoma [45,46]. The survey prompt and questionnaire items are provided in Multimedia Appendix 1. The influence of several predictors was examined through the conceptual model developed for the study: (1) performance expectancy, (2) effort expectancy, (3) social influence, (4) perceived risk, (5) trust, and (6) trialability. The first 5 constructs were derived from the UTAUT model [45]. The sixth construct, trialability, was derived from the DOI model [47,48]. The 6 constructs from the UTAUT and DOI frameworks were the independent variables for this study.

The final survey was adapted from the UTAUT questionnaire items developed by Venkatesh et al [45]. Two of the researchers (RC and SF) developed, pilot tested with 4 respondents not involved in the study, and refined the survey (eg, question order and wording) before it was administered to the study sites. The survey used in this study contained statements related to each construct. Respondents expressed their level of agreement with each statement using a 7-point Likert scale, with 1 being *Strongly agree* and 7 being *Strongly disagree*. The constructs within the conceptual model have been found to have high reliability and validity in previous studies: standardized factor loadings and average variance extracted values exceeded the recommended threshold of 0.50 [46,51,52]. Furthermore, the composite reliability values were above 0.90 for each of the constructs, surpassing the minimum recommended value of 0.70 [46,53]. Similar findings have emerged for the behavioral intention construct [46]. Preliminary analysis of the survey data collected for this study also met the recommended thresholds for reliability and validity.

An initial prompt to participate in the survey was sent via email. The prompt briefly described the study (eg, purpose, procedures, risks, and benefits). In addition, the prompt included a link to the web-based survey and a consent form that provided more details about the study. Data collection began on June 4, 2018, and ended on August 14, 2018.

#### Variables

*Behavioral intention* was the outcome of the combination of the independent variables. It was also a predictor of use behavior. Similar to the independent variables, behavioral intention was operationalized as an ordinal-level variable. Survey items related to behavioral intention were answered using the same 7-point Likert scale adopted for the independent variables.

*Use behavior* was operationalized as a dichotomous variable. Participants answered *Yes* or *No* to the initial screening question: “Have you ever used behavioral health information obtained from the HIE your organization uses?” Participants who responded *Yes* answered questions related to performance expectancy, effort expectancy, social influence, perceived risk, and trust. Participants who responded *No* answered an additional set of questions pertaining to trialability. On the basis of the response to the initial screening question, the wording of the subsequent questions was modified to reflect actual experiences versus perceptions of obtaining behavioral health information from the HIE.

Finally, the survey instrument included questions designed to collect demographic information, which included data related to gender, education, job title, age, race or ethnicity, and level of comfort and experience with technology. This study was conducted with the approval of the University of Alabama at Birmingham IRB #300001017.

#### Data Analysis

For the survey data, preliminary descriptive statistics were generated. Furthermore, we tested the reliability and validity of the survey items. We used partial least squares structural equation modeling (PLS-SEM) to test the relationships between the constructs in the conceptual model. PLS-SEM is a nonparametric alternative to covariance-based structural equation modeling, which makes it more applicable to this study, as we have a small sample and data that are not normally distributed [54-57]. PLS-SEM can be used in both exploratory and confirmatory studies [58]. All statistical analyses were performed using Stata version 15 at an α level of .05.

### Qualitative Phase

#### Sampling

Semistructured interviews were conducted with a subsample of the survey participants to better understand the role of these factors from the subjects’ perspectives and to discover any additional factors that were not captured in the survey. A total of 18% (11/62) survey participants provided their contact information for a follow-up interview at the end of the quantitative survey. Of these, 5 completed the interviews.

#### Interview Guide

Three of the authors (RC, SF, and NI) collaborated on the development of the interview guide for this phase. The interview questions were developed to elaborate on predictors of intention to use behavioral health information from an HIE that emerged from the initial quantitative survey. Therefore, the interview guide was designed to facilitate a closer examination of the constructs from the UTAUT and DOI frameworks. Likewise, the interview questions also captured information about findings that were contradictory to previous research to better understand the emergence of these inconsistencies [59]. Before deployment, the interview guide was pilot tested with 4 individuals with expertise in health informatics (n=3) and psychiatric nursing (n=1). Minor revisions were made to the wording and ordering of the interview questions. Multimedia Appendix 2 provides the final interview guide.

### Data Collection

The first author (RC) conducted the interviews with the survey respondents. An initial request for interview email was sent to the participants, and follow-up emails were sent 2 and 4 weeks after the initial request. Interviews were conducted either in person or via teleconference (Zoom), based on interviewee preference and timing. All interviews were recorded and transcribed using the Otter Voice Notes mobile app. The interviews were conducted from March 19 to May 2, 2019.

### Data Analysis

Verbatim interview transcripts were analyzed using inductive and deductive thematic analysis with NVivo 12. The development of the interview questions was partially guided by the constructs in the conceptual model; additional themes were derived from the content within the interview transcripts [60]. The following 4-stage analytical process was adopted for the study: (1) becoming familiar with data, (2) generating themes by aggregating coded segments of data, (3) creating comparative categories, and (4) revising and refining the themes [61]. Two of the authors (RC and SF) engaged in peer debriefing to discuss the interview process and the resulting themes, thus generating additional insights into the interview findings [62]. To increase the trustworthiness of data, we used member checking: participants received a 1- to 2-page summary of the interview transcript from the interviewee (RC) by email and were asked to provide clarification or ask additional questions. Of the 5 participants, 2 responded to the follow-up email. One participant provided additional clarification for one of the discussion points; the other participant found no errors in the summary document. Quantitative and qualitative findings were further integrated to create meta-inferences using a joint display [63].

## Results

Due to the sequential nature of this mixed methods study design, we present the results in sequence: quantitative findings followed by qualitative findings and then followed by the integration of quantitative and qualitative findings.

### Quantitative

#### Descriptive Statistics

Descriptive statistics of the survey participants are presented in Table 1. A total of 90% (56/62) of the respondents reported that they did not exchange behavioral health data via the HIE. A majority of the respondents (49/62, 79%) were female, and 83% (52/62) of the respondents were aged between 30 and 59 years. A large proportion of the health care providers in this sample were identified as nurses (27/62, 44%). Nonclinical roles (23/62, 37%) consisted of directors, network administrators, and patient experience representatives. With regard to the reported level of computer experience, most participants (45/62, 72%) identified as *average* users.

**Table 1:** Descriptive statistics of behavioral health information exchange survey participants (N=62).

Variables			Values, n (%)
**Retrieved behavioral health data from their organization’s HIE^a^**			
	Yes	6 (10)	
	No	56 (90)	
**Gender**			
	Female	49 (79)	
	Male	12 (19)	
	Prefer not to state	1 (2)	
**Age group (years)**			
	21-29	3 (5)	
	30-39	18 (29)	
	40-49	18 (29)	
	50-59	16 (25)	
	60-69	6 (10)	
	≥70	1 (2)	
**Job title**			
	Nurses	27 (44)	
	Physicians	10 (16)	
	Physician’s assistants	2 (3)	
	Others	23 (37)	
**Education**			
	High school diploma or GED^b^	3 (5)	
	Some college	8 (13)	
	2-year degree	20 (32)	
	4-year college degree	10 (16)	
	Master’s degree	10 (16)	
	Professional degree	10 (16)	
	Doctorate	1 (2)	
**Hispanic or Latino origin**			
	Yes	3 (5)	
	No	59 (95)	
**Race**			
	American Indian or Alaska Native	2 (3)	
	Asian	5 (8)	
	Black or African American	6 (10)	
	White	47 (76)	
	Other	2 (3)	
**Level of computer experience**			
	Novice	1 (2)	
	Average	45 (73)	
	Advanced	16 (26)	

^a^HIE: health information exchange.

^b^GED: general education development.

#### Measurement Validity

The measurement model determined the reliability and validity of the survey items associated with each latent construct in the conceptual model. Cronbach α scores of .7 or higher indicate internal consistency [64,65]. The measurement model is illustrated in Multimedia Appendix 3. With the exception of one item on the performance expectancy construct, all items met the criteria for internal consistency.

#### PLS-SEM Results

The results of PLS-SEM are presented in Table 2. The standardized path coefficients (β) denote the strength and direction of the relationship between the constructs in the conceptual model.

**Table 2:** Path analysis of the constructs within the conceptual model.

Predictors of intention to use behavioral health information from the HIE^a^	*β* ^b^	*P* value
Performance expectancy	.382	.01
Effort expectancy	.055	.72
Social influence	−.043	.72
Perceived risk	.061	.47
Trust	.539	<.001
Trialability	.093	.34
Use behavior	.127	.68

^a^HIE: health information exchange.

^b^ß represents the standardized path coefficients from the partial least squares structural equation modeling results. The standardized path coefficients indicate the strength and direction of the relationship between the constructs.

The PLS-SEM results revealed that performance expectancy and trust are the significant predictors of behavioral intention to exchange behavioral health information via HIEs. On average, survey participants who reported higher scores on the performance expectancy measures also reported higher scores on the behavioral intention measures (β=.383; *P*=.01). Similarly, participants who scored higher on the trust measures also had higher scores on the behavioral intention measures (β=.539; *P*<.001). In this study, trust was found to be the strongest predictor of behavioral intention. Furthermore, behavioral intention was not significantly associated with the actual use of behavioral health information from HIEs.

### Qualitative

#### Interview Participants and Overview

Of the 5 individuals who participated in the interviews, only 1 was employed in Oklahoma. The majority of the participants were nonclinical employees, with only 1 participant (a nurse practitioner) involved in direct patient care. The length of the interviews ranged from 19 minutes to 60 minutes, with an average interview length of 34 (SD 16) minutes.

#### Themes From the Interviews

A total of 6 themes emerged from the interviews: (1) usefulness of behavioral health information in care delivery, (2) regulations restricting behavioral HIE, (3) behavioral health and stigma, (4) missing or difficult-to-locate behavioral health data, (5) lack of mandatory training for behavioral HIE, and (6) future utilization of the HIEs. Each theme is explored in detail in the following sections. More details about each theme and the related participants’ quotes are given in Multimedia Appendix 4.

##### Usefulness of Behavioral Health Information in Care Delivery

Sharing behavioral health information can improve care delivery. In various settings, exchanging health data via an HIE increases efficiency. More specifically, exchanging behavioral health information in an emergency department could help the attending physician to become familiar with a patient’s medical history and could be used in discharge planning. Despite these perceived benefits, there are several barriers that prohibit the exchange of behavioral health information.

##### Regulations Restricting Behavioral HIE

Existing regulations and policies make behavioral HIE challenging. One of the most significant perceived barriers to behavioral HIE, 42 CFR Part 2, makes health care workers hesitant to share sensitive information electronically. 42 CFR Part 2 leaves room for interpretation with regard to the exchange of behavioral health information, and it is not uncommon for health care administrators to err on the side of caution.

Overall, the participants expressed doubt that behavioral HIE will become a common practice in the near future, given the influence of 42 CFR Part 2. The restrictions surrounding the exchange of behavioral health information will most likely discourage the sharing of this information, unless it is deemed essential to the provision of care. Even with the recent relaxation of 42 CFR Part 2, one interviewee specifically stated that behavioral health data will never be effectively shared via HIE: “[42 CFR Part 2] simply discourages it.” 42 CFR Part 2 was enacted as a response to the stigma surrounding sensitive diagnoses, including those related to behavioral health. This stigma persists even today.

##### Behavioral HIE and Stigma

Stigma continues to surround behavioral health conditions and contributes to the suppression of behavioral health information in medical records. As such, mental illness continues to be considered taboo, and because of this, information related to a mental health diagnosis is often separated from the rest of a patient’s medical record. Furthermore, the existing stigma may discourage patients from revealing their diagnoses to a new health care provider. This hesitation to disclose this information hinders the flow of health-related information and complicates the delivery of appropriate care, especially if the provider is unfamiliar with the patient’s medical history.

##### Missing or Difficult-to-Locate Behavioral Health Information

One of the concerns raised about incorporating behavioral health information into the electronic medical record was centered on locating the information within the HIE. One interviewee suggested that if behavioral health information is difficult to identify, it could reduce efficiency in care provision. In addition, removing behavioral health patients’ records from the HIE further complicates the delivery of appropriate care.

General medical practitioners who access the HIE do not specifically look for behavioral health information. As such, participants do not know where this information would be located within the patient records. A participant (interviewee 4) suggested that it might be beneficial to provide training to assist health care providers in identifying behavioral health information in the patient record. Adequate training could not only reduce the potential workflow issues associated with behavioral HIE but could also facilitate the provision of appropriate care.

##### Lack of Mandatory Training for Behavioral HIE

The local network administrator for the Alabama site (interviewee 2) trained participants on the proper use of the HIE. There was no component of the training that focused exclusively on the exchange of behavioral health information via the HIE. The training encompassed basic functions (eg, how to log in, what information can be found within the system, and where to locate it). The training provided was not mandatory. In fact, some participants learned about HIEs via word of mouth. One participant (interviewee 4) believed that providing formal training could allow care providers to provide input regarding the usefulness of clinical data. Likewise, the training could help them to understand why there are restrictions on exchanging certain types of clinical data, such as behavioral health information.

The lack of mandatory training for HIE use made it challenging for providers to figure out how to integrate behavioral health information. However, the interviewees expressed hope that they will be able to increase the utilization of HIEs in the future.

##### Future Utilization of HIEs

The interviewees acknowledged the value of exchanging general health data across HIEs and hoped to increase their utilization in the near future. Some participants also saw value in using the systems to exchange behavioral health information between multiple providers, despite the numerous barriers that currently exist. One participant (interviewee 4) suggested that these HIEs might be necessary to provide effective treatment in appropriate care settings to these difficult-to-treat patients.

### Integration of the Quantitative and Qualitative Findings

We further integrated the quantitative and qualitative findings using a joint display. The joint display enabled us to compare the quantitative and qualitative results, explain similarities and differences between the findings, and develop meta-inferences regarding the exchange of behavioral health information via an HIE (Multimedia Appendix 5). In summary, the participants stated that having access to a patient’s full medical record, including behavioral health diagnoses, would be useful in delivering appropriate treatment. Despite the potential benefits of having access to this information, there are several barriers that prohibit the exchange of behavioral health information. These barriers are primarily related to technical capabilities, policies and regulations, stigma, and training. Even with these challenges, the interviewees acknowledged the value of having complete information and expressed some hope that behavioral HIE will become standard practice in the future.

## Discussion

### Principal Findings

The purpose of this mixed methods study is to identify and describe factors that may influence the intention to exchange and use behavioral health information obtained from an HIE. The preliminary findings from the quantitative phase of the study indicate that performance expectancy and trust are significant predictors of behavioral intention to exchange behavioral health information via HIEs. In other words, participants believed that exchanging behavioral health information via HIEs would improve their job performance. However, 90 % (56/62) of survey participants reported that they did not use HIEs to exchange behavioral health information, which likely contributed to the insignificant association between behavioral intention and actual use. The misalignment between performance expectancy and behavioral intention is supported in the literature [66,67]. One explanation for this finding is that understanding the potential job-related benefits of using a technology might not motivate health care professionals to adopt it if there are other contextual factors to consider [67].

Within health care, trust is a predictor of the intention to use technology among both patients and physicians [68,69]. Our findings were consistent with the literature in that participants suggested that the lack of full disclosure of behavioral health conditions, possibly due to stigma, may produce an incomplete record and not provide practitioners with valuable clinical information at the point of care.

In summary, 2 constructs (performance expectancy and trust) emerged as significant predictors in the initial quantitative phase of the study. One theme (usefulness of behavioral health information in care delivery) emerged from qualitative interviews that provided further insights into the significance of the performance expectancy construct. However, there were several divergent findings. Perceived risk did not emerge as a significant predictor in the quantitative phase; however, the interviewees stated that regulations and stigma are barriers to behavioral HIE. The interviewees suggested some connection between trust and stigma of behavioral health diagnoses. One interviewee stated that people were hesitant to disclose behavioral health information; therefore, the information that could truly be helpful at the point of care is often missing in terms of behavioral health (interviewee 1). Trialability did not emerge as a significant predictor of behavioral intention in the quantitative phase; however, the interviewees discussed a lack of in-depth training in the subsequent qualitative phase. Finally, behavioral intention was not a significant predictor of the actual use of HIEs for the purpose of behavioral HIE. This is likely due to the fact that participation in behavioral HIE efforts at these 2 study sites is low. However, interviewees expressed hope that in the future, HIEs will be used to exchange the full patient record, including any behavioral health diagnoses or treatment.

### Implications and Future Research

As behavioral health becomes a growing public health concern, health care practitioners have largely accepted the need for integrated care [70,71]. Early integrated care models have shown improvements in patients’ mental and physical health [71-74]. In addition, health care providers have expressed the belief that omitting behavioral health data from patient records hinders their ability to make appropriate treatment recommendations [71]. The findings of this study provide additional support to these notions.

The successful delivery of integrated care requires a reconsideration of existing policies and regulations as well as the establishment of clear guidelines and best practices for handling sensitive health-related information [75]. In the wake of the COVID-19 pandemic, restrictions on the sharing of personal health information have been relaxed. The requirement to obtain patient consent may be waived if the provider determines that there is a bona fide medical emergency [76]. It is crucial for stakeholders to consider whether this regulatory change should be retained following the pandemic.

### Limitations

Although the findings are valid in the context of this study, the small sample size may limit its generalizability. This is a common limitation of studies that are conducted during early uses of IT, thus necessitating the use of nonparametric methods such as PLS-SEM [77]. With the exchange of behavioral health data via HIE being relatively nascent (at the time of this study), this limitation is expected. Furthermore, the relationships between these constructs cannot be deemed causal but rather provide some insights into perceptions and reality. Similarly, because there were few participants who exchanged behavioral health information via HIEs, there was not enough variation in the responses to determine whether there were different sets of predictors for those who used the HIEs to exchange behavioral health information and those who did not. It is possible that the use of convenience sampling contributed to the homogeneity in the responses. However, the lack of active participants in behavioral HIE efforts could also be partially explained by the novelty of both the Alabama and Oklahoma HIEs.

Even in light of these limitations, the findings provide early positive suggestions for the use of behavioral health data shared by HIEs. This study is the first to adopt a mixed methods approach to examine the use of HIEs to share patients’ behavioral health information. The use of mixed methods allowed the researchers to identify and explore factors that may influence the adoption and use of HIEs to facilitate behavioral HIE. This study was conducted across multiple states. Finally, the study is applicable to both the health services and health IT arenas. It is relevant to the health services literature because it acknowledges the importance of a whole-person approach to care. Likewise, it is relevant to health IT because it considers the role of technology in delivering integrated care to patients with complex needs.

### Conclusions

Patients with behavioral health conditions are readmitted to the hospital 30 days after discharge at a higher rate than patients without behavioral health disorders. Several factors contribute to this pattern of overutilization. Existing regulations have traditionally restricted the exchange of behavioral health information unless the patient has given authorization at the person level, meaning that the specific person must be identified. The prevalence of suboptimal treatment outcomes for behavioral health patients with chronic illnesses indicates that it is necessary to commit more efforts to providing higher quality care to some of the most vulnerable patients.

Health care providers acknowledge that it is necessary to deliver holistic care to improve the quality of care and health-related outcomes for this subset of difficult-to-treat patients. This study contributes to our understanding of the potential role of HIEs in integrating the traditionally fragmented behavioral and general health service arenas.

## Appendix

Multimedia Appendix 1 Survey prompt and questionnaire items.

Multimedia Appendix 2 Interview questions.

Multimedia Appendix 3 Measurement model.

Multimedia Appendix 4 Themes and quotes from qualitative interviews (phase 2).

Multimedia Appendix 5 Joint display of quantitative and qualitative findings with meta-inferences.

## Abbreviations

CFR: Code of Federal Regulations
DOI: diffusion of innovation
HIE: health information exchange
IT: information technology
PLS-SEM: partial least squares structural equation modeling
UTAUT: unified theory of acceptance and use of technology
