# Supplementary material for: Intention to Use Behavioral Health Data From a Health Information Exchange: Mixed Methods Study
Source: JMIR Ment Health. 2021 May 27;8(5):e26746. doi: 10.2196/26746 (PMC8193493; doi:10.2196/26746)
Supplement: Multimedia Appendix 1 [file mental_v8i5e26746_app1.docx]

**Multimedia Appendix 1. Survey prompt and questionnaire items.**

Hello, and thank you for taking time to complete this survey. The survey will be used to determine factors that influence the intention to use behavioral health information obtained from a health information exchange. The survey is to be completed by physicians, nurse practitioners, and other healthcare employees who obtain and use health information from the organization’s HIE.

Have you ever used behavioral health information obtained from the HIE your organization uses? __ Yes __ No

**Performance Expectancy**

- P1. I would find the use of behavioral health information obtained from HIE useful in my job.
- P2. Using behavioral health information obtained from HIE enables me to accomplish tasks more quickly.
- P3. Using behavioral health information obtained from HIE increases my productivity.
- P4. If I use behavioral health information obtained from HIE, I will increase my chances of timely reimbursement.

**Effort Expectancy**

- E1. My interaction with behavioral health information obtained from HIE would be clear and understandable.
- E2. It would be easy for me to become skillful at using behavioral health information obtained from HIE.
- E3. I would find it easy to use behavioral health information obtained from HIE.
- E4. Learning to obtain behavioral health information from HIE is easy for me.
- E5. I have no difficulty finding behavioral health information within the information obtained from the HIE.
- E6. I have no difficulty understanding the behavioral health information that I obtained from the HIE.

**Social Influence**

- S1. People who influence my behavior think I should use behavioral health information obtained from HIE.
- S2. People who are important to me think that I should use behavioral health information obtained from HIE.
- S3. The senior management of this organization has been helpful in the use of behavioral health information obtained from HIE.
- S4. In general, the organization has supported the use of behavioral health information obtained from HIE.

**Perceived Risk**

- PR1. I do not feel totally safe providing patients’ behavioral health information through HIE.
- PR2. I am worried about using behavioral health information obtained from HIE because other people may be able to access patient information.
- PR3. I do not feel secure sending patients’ behavioral health information through HIE.

**Trust**

- TRU1. I trust the behavioral health information received through HIE.
- TRU2. I trust the behavioral health information received through HIE to be reliable.
- TRU3. I trust the behavioral health information received through HIE to be secure.
- TRU4. I believe the behavioral health information received through HIE is trustworthy.

**Trialability**

- TRI1. I would be more likely to want to obtain and use behavioral health information from HIE if I were able to participate in a pilot test.
- TRI2. I really won’t lose much by trying to obtain and use behavioral health information from HIE, even if I don’t like it.

**Behavioral Intention to Use the Information**

- B1. I intend to use behavioral health information obtained from HIE.
- B2. I predict that I will use behavioral health information obtained from HIE.
- B3. I plan to use behavioral health information obtained HIE.

**Demographic Information**

1. What is your gender? __Male __Female __Prefer not to state
2. What is the highest level of education you have completed?

__High school/GED

__Some college

__2-year college degree (Associate)

__4-year college degree (BA, BS, BSN, etc.)

__Master’s degree (MA, MS)

__Professional degree (MD, PharmD)

__Doctoral degree (PhD, DNP, etc.)

1. How old are you?

__21-29

__30-39

__40-49

__50-59

__60-69

__70+

1. Are you of Hispanic or Latino origin? __Yes __No
2. What is your racial background? (Check all that apply)

__American Indian/Alaska Native

__Asian

__Native Hawaiian or Other Pacific Islander

__Black/African American

__White

__Other (please specify): __________

1. Which best describes the level of your computer skills?

__Novice user (You just started using computers)

__Average user (You use word processors, spreadsheets, e-mail, surf the Web, etc.)

__Advanced user (You can install software, setup configurations, etc.)

__Expert user (You can setup operating systems; know some computer programming languages, etc.)

1. How many years of computer experience do you have? ___ years
2. Do you feel that some of your computer use has been replaced by use of a tablet or smartphone? __Yes __No
3. By what percentage would you estimate that your computer use has been replaced by the use of a tablet or smartphone?
4. Please write any additional comments that you may want to share.
5. This survey is anonymous. However, if you would like to be contacted for an interview, please provide your contact information below.
