# Supplementary material for: Intention to Use Behavioral Health Data From a Health Information Exchange: Mixed Methods Study
Source: JMIR Ment Health. 2021 May 27;8(5):e26746. doi: 10.2196/26746 (PMC8193493; doi:10.2196/26746)
Supplement: Multimedia Appendix 2 [file mental_v8i5e26746_app2.docx]

**Multimedia Appendix 2. Interview questions.**

**Section 1: Introductory Questions**

1. Please tell me about yourself and your role in the organization.
2. Please describe your role/interaction with the HIE in your organization.
3. Please describe how HIEs are used within your organization.
4. Could you describe for me under what conditions you have or would use the HIE to retrieve behavioral health information?

**Section 2: Behavioral Health Information Exchange Initiative**

1. The survey suggests that the query and use of behavioral health information via the HIE is low. What are your thoughts on this finding?
2. What do you think your organization can do to increase the query and use of behavioral health information through the HIE?

**Section 3: Performance Expectancy**

1. In your opinion, how can behavioral health information query and use be utilized in delivering care to patients?
2. In your opinion, what challenges might arise for healthcare providers as a result of querying and using behavioral health information through the HIE?

**Section 4: Trust**

1. How has the exchange of general health information been useful to you in treating patients?
2. In your opinion, what would make general health information more useful in treating patients?
3. Now, let’s think about behavioral health information. What are your thoughts about the usefulness of behavioral health information **specifically** in treating patients?

**Section 5: Trialability**

1. Please describe any opportunities the organization provided to learn about exchanging behavioral health information.
2. If your organization did not provide the opportunity to participate in a pilot test, in what ways do you think a pilot test could facilitate behavioral health information exchange?

**Section 6: Effort Expectancy**

1. Please describe the process of querying and using behavioral health information from the HIE, as you currently understand it.
2. What challenges, if any, do you think you would face in querying and using behavioral health information from the HIE?
3. How would these challenges impact your ability to treat patients?

**Section 7: Perceived Risk**

1. What are your perceptions of behavioral health information?
2. What are some of the potential risks of sharing behavioral health information electronically?

**Section 8: Additional Factors/Concluding Thoughts**

1. What else would you like to discuss regarding the query and use of behavioral health information via the HIE?
2. May I have your permission to reach back out to you to follow up on any of the points that you made today, if necessary?
