# Supplementary figures and images for: Intention to Use Behavioral Health Data From a Health Information Exchange: Mixed Methods Study
Source: JMIR Ment Health. 2021 May 27;8(5):e26746. doi: 10.2196/26746 (PMC8193493; doi:10.2196/26746)

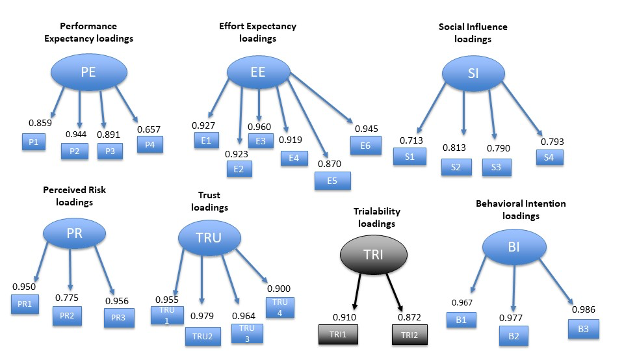

Supplement: Multimedia Appendix 3 [file mental_v8i5e26746_app3.png]
