# Supplementary material for: Intention to Use Behavioral Health Data From a Health Information Exchange: Mixed Methods Study
Source: JMIR Ment Health. 2021 May 27;8(5):e26746. doi: 10.2196/26746 (PMC8193493; doi:10.2196/26746)
Supplement: Multimedia Appendix 4 [file mental_v8i5e26746_app4.docx]

**Multimedia Appendix 4. Themes and quotes from qualitative interviews (phase 2).**

| Theme | Description of Theme | Illustrative Quote |
| --- | --- | --- |
| Usefulness of behavioral health information in care delivery | Interviewees detail the ways in which BHIE can improve delivery/provision of care in healthcare settings. Likewise, they talk about the ways in which incorporating behavioral health information into the patient’s medical record can be challenging. | “A lot of patients come in and don’t know exactly what all their diagnoses are, what all their medications are… That information tends to be vitally important to us… if for no other reason, giving us a historic perspective about what the… patient’s baseline presentation is and diagnoses are… as well as… addressing acute issues.” (Interviewee 4) |
| Regulations restricting the exchange of behavioral health information | Interviewees highlight some of the regulatory barriers that complicate the exchange of behavioral health information. | “The priority is making sure that we are… in the clear when it comes to [regulations such as] 42 CFR Part 2, you know? The priority is not ensuring that behavioral and mental health information is able to make it to the next provider on the HIE.” (Interviewee 2) |
| Behavioral health information exchange and stigma | Interviewees discuss the stigma that continues to surround behavioral health and how it complicates the exchange of behavioral health information. | “You know, behavioral health stuff… there’s a lot of taboo tied to what’s going on with people… It’s a little bit more challenging to access that information.” (Interviewee 4) |
| Missing or difficult-to-locate behavioral health information | Interviewees discuss how much effort they believe they’d have to put forward to learn how to exchange/identify behavioral health information within a patient record. | “When I pull [the system] up, I look at the whole clinical dashboard. I’m not even recalling if I’ve seen, like, a particular place where it’s only talking about their behavioral background.” (Interviewee 3) |
| Lack of mandatory training for behavioral health information exchange | Interviewees discuss whether they participated in a pilot test and what they learned, or they discuss the ways in which they think they could have benefited from a pilot test. | “I don’t know if there’s any training or education… that specifically had to do with behavioral health information…” (Interviewee 2) |
| Future utilization of the HIEs | Interviewees share concluding thoughts about the use of the HIEs for general medical and behavioral health information exchange. | “…I’m hopeful that in the future… exchanging behavioral and mental health information will… as an industry… that we’ll kind of shift more to the… full exchange of that information.” (Interviewee 2) |
