# Supplementary material for: Intention to Use Behavioral Health Data From a Health Information Exchange: Mixed Methods Study
Source: JMIR Ment Health. 2021 May 27;8(5):e26746. doi: 10.2196/26746 (PMC8193493; doi:10.2196/26746)
Supplement: Multimedia Appendix 5 [file mental_v8i5e26746_app5.docx]

**Multimedia Appendix 5. Joint display of quantitative and qualitative findings with meta-inferences.**

| Variables | Quantitative, path coefficient | *P* value | Qualitative | | Meta-inferences (relationship between quantitative constructs and qualitative themes) |
| --- | --- | --- | --- | --- | --- |
| Construct |  |  | Theme | Illustrative quotes |  |
|  | |  | | | |
| Performance expectancy | .382 | .01 | Usefulness of behavioral health information in care delivery | - “...if you don’t know somebody...and you’re having to see another provider...that can help them understand too...Maybe there’s something...mental status that says, ‘This is why’.” - “I think the HIE...[is] very valuable in [the emergency department]...they’ve never seen this patient before probably. And they can view the HIE and get a...broad look at a health history for this patient...mental and behavioral health...diagnoses that may affect how they want to treat...or interact with that patient, and kind of their bedside manner...within the emergency department.” - “If I have a patient that comes in with either altered level of consciousness, or they come in with a drug overdose, or...some kind of acute confusion going on...it would be helpful to know if there was any pre-existing...mental health issue that required medications that could have instigated that situation.” | The “usefulness” theme provides further insights into the significance of performance expectancy. Having patients’ full medical history (including behavioral health diagnoses and treatment) is helpful, particularly in the emergency department where the physician may be encountering the patient for the first time. |
| Effort expectancy | .055 | .72 | Missing or difficult-to-locate behavioral health information | - “Okay, the information may be there, but where does it appear for the doctor? ...Does this fit into their workflow? ...an HIE is only as good as, you know, its providers are at using it, so it can’t have...an impact on patient care or patient outcomes unless...the providers or nurses or front office staff are actually utilizing and putting to good use.” - “[The system], I think...accesses some general discharge information, admission and discharge information...I don’t recall seeing anywhere where there was...listings of medications there. Again, we have that separate somewhere else in our system.” - “When I pull [the system] up, I look at the whole clinical dashboard. I’m not even recalling if I’ve seen, like, a particular place where it’s only talking about their behavioral background.” | Effort expectancy did not predict intention to exchange behavioral health information via the HIEs. However, interviewees expressed concern that behavioral health information would be difficult to locate in the HIE, thus suggesting that learning to identify and exchange this information would require additional effort from the provider. |
| Social influence | −.043 | .72 | No themes related to social influence emerged from the interviews | No quotes related to social influence emerged from the interviews. | Social influence did not predict intention to exchange behavioral health information via HIEs. The interviewees did not discuss efforts from supervisors to encourage behavioral health information exchange. Therefore, no themes or quotes related to social influence emerged. |
| Perceived risk | .061 | .47 | Regulations restricting the exchange of behavioral health information | “The priority is making sure that we are...in the clear when it comes to [regulations such as] 42 CFR Part 2, you know? The priority is not ensuring that behavioral and mental health information is able to make it to the next provider on the HIE.” | Perceived risk did not predict intention to exchange behavioral health information via HIEs, but the interviewees acknowledged regulations (eg, 42 CFR Part 2) that restrict the exchange of this information. The organization could risk facing fines if these regulations are violated. |
| — | — | — | Behavioral health information exchange and stigma | - “You know, behavioral health stuff...there’s a lot of taboo tied to what’s going on with people...It’s a little bit more challenging to access that information. People are a lot less likely to just release that information.” - “I think you could kind of make the case that...the long-standing stigma that our society has had against, you know, mental health issues or behavioral health issues...kind of manifests itself in...42 CFR or other...regulatory captures.” | The interviewees identified a second level of perceived risk: risk to the patient. As behavioral health disorders are highly stigmatized, patients fear the possibility of persecution and/or loss of employment if this information is released. |
| Trust | .539 | <.001 | No themes related to trust emerged from the interviews | No quotes related to trust emerged from the interviews. | Trust predicted intention to exchange behavioral health information via HIEs. The interviewees did not express beliefs that behavioral health information is less trustworthy than general medical information; therefore, it did not emerge as a theme in the qualitative phase of the study. |
| Trialability | .093 | .34 | Lack of mandatory training for behavioral health information exchange | - “No, there wasn’t [any training]...I think if we had a pilot test, you know, we could have provided some input on things that would have been helpful with regard to what we...would like to be in there, or have access to...we could understand why [certain] things aren’t accessible.” - “I don’t know if there’s any training or education...that specifically had to do with behavioral health information, but...when a new member organization joins the HIE...that’s part of my job...is the training and activation part of it.” - “...he did a training for us...I mean, mostly, it was just knowledge of how to get [the information] we needed to get...It was more of a physically using the website kind of training.” | Trialability did not predict intention to exchange behavioral health information via HIEs. The training that was provided focused on basic information related to the HIEs, and it was not mandatory. No aspect of the training focused on exchanging and obtaining behavioral health information from HIEs. |
| Behavioral intention | .127 | .68 | Future utilization of HIEs | - “...I’m hopeful that in the future...exchanging behavioral and mental health information will...as an industry...that we’ll kind of shift more to the...full exchange of that information. ...I think it should be just a part of the patient’s whole medical record and, you know, when you’re holistically treating a patient, you need to know about any mental or behavioral health diagnoses or issues they may have.” - “...I’m hopeful that with putting systems in place...in the hospitals, as well as a resurgence and then investing in open facilities to keep these patients, to take care of them, providing the care they need.” - “...I think now that I know [the system is] operational, we’ll be able to use it more...like I said, I didn’t even know it was working ‘til about two weeks ago.” | Behavioral intention did not predict the actual use of HIEs to exchange behavioral health information. This finding is likely because of the fact that participation in behavioral health exchange efforts is low. However, the interviewees expressed some hope that in the future, it will become standard practice to share patients’ behavioral health records. |

^a^—: No additional data available.
